# Effects of altered sagittal trunk orientation on kinetic pattern in able-bodied walking on uneven ground

**DOI:** 10.1242/bio.025239

**Published:** 2017-05-30

**Authors:** Soran Aminiaghdam, Christian Rode

**Affiliations:** Department of Motion Science, Institute of Sport Sciences, Friedrich Schiller University Jena, Seidelstraße 20, Jena 07740, Germany

**Keywords:** Locomotion, Posture, Kinetics, Ground reaction force

## Abstract

Studies of disturbed human locomotion often focus on the dynamics of the gait when either posture, movement or surface is perturbed. Yet, the interaction effects of variation of trunk posture and ground level on kinetic behaviour of able-bodied gait have not been explored. For 12 participants we investigated the kinetic behaviour, as well as velocity and contact time, across four steps including an unperturbed step on level ground, pre-perturbation, perturbation (10-cm drop) and post-perturbation steps while walking with normal speed with four postures: regular erect, with 30°, 50° and maximal sagittal trunk flexion (70°). Two-way repeated measures ANOVAs detected significant interactions of posture×step for the second peak of the vertical ground reaction force (GRF), propulsive impulse, contact time and velocity. An increased trunk flexion was associated with a systematic decrease of the second GRF peak during all steps and with a decreased contact time and an increased velocity across steps, except for the perturbation step. Pre-adaptations were more pronounced in the approach step to the drop in regular erect gait. With increased trunk flexion, walking on uneven ground exhibited reduced changes in GRF kinetic parameters relative to upright walking. It seems that in trunk-flexed gaits the trunk is used in a compensatory way during the step-down to accommodate changes in ground level by adjusting its angle leading to lower variations in centre of mass height. Exploitation of this mechanism resembles the ability of small birds in adjusting their zig-zag-like configured legs to cope with changes in ground level.

## Abstract (precis)

**Summary:** In able-bodied trunk-flexed gaits, adjustment of trunk kinematic may facilitate negotiation of changes in ground level (10-cm drop and pre/post steps), as reflected in more consistent ground reaction force parameters.

## INTRODUCTION

On the one hand, the negotiation of changes in the surface such as compliance, slip, obstacle or drop during walking challenges the human locomotor system and requires continuous adaptations (Tang et al., 1998; Marigold and Patla, 2002, 2005, 2008; van Dieen et al., 2007; Shinya et al., 2009; Müller et al., 2014). On the other hand, the generation of the ground reaction force (GRF) in human walking is strongly influenced by the orientation of the trunk (50% of total human body mass) owing to its significant effect on the displacement and acceleration of the body centre of mass (CoM) (Grasso et al., 2000; Gillet et al., 2003; Marigold and Patla, 2005; Saha et al., 2008; Leteneur et al., 2009; Kluger et al., 2014; Aminiaghdam et al., 2017).

Understanding changes in gait dynamics and accompanying compensatory techniques under both internal (posture) and/or external (surface) perturbations can shed light into functional demands of bipedalism in various scientific areas. For example, improved knowledge of the role of the trunk orientation in gait is of clinical interest as age or some pathological conditions alter trunk posture and adaptive capacity of the locomotor system (Farcy and Schwab, 1997; Lin et al., 2000; Sarwahi et al., 2002; Potter et al., 2004; Malone et al., 2015). Furthermore, the study of human gait with a crouched posture, i.e. mimicking pronograde locomotion of birds is of interest for comparative biologists (Gatesy and Biewener, 1991; Hirasaki et al., 2004; Schwartz, 2007; Thorpe et al., 2007; Foster et al., 2013; Aminiaghdam et al., 2017). In addition, experimental studies focused on investigating how human anatomy performs in different locomotor postures may provide further explanation for interpretation of the evolution of human bipedal locomotion. In general, exploration of gait features in a setting with greater variations of posture or ground level may also elicit the functional demands that have influenced the evolution of human bipedalism better than walking on uniform surfaces (Sockol et al., 2007; Pontzer et al., 2009).

Balancing the trunk, basically the functional task of stabilising an unstable inverted pendulum standing on the hip (Maus et al., 2010), plays an important role in human locomotion. The trunk has been suggested to serve as a reference in the control of posture and movement (Mouchnino et al., 1993; Darling and Miller, 1995; Massion et al., 1997). Furthermore, a forwardly bent trunk induces a gravitational moment that can be utilised to generate greater forward propulsion through the hip (Leroux et al., 2002) which in turn facilitates walking uphill/climbing stairs or to accelerate. At the same time, because the trunk is heavy, a forward bent trunk allows vertical alteration of CoM height (Aminiaghdam et al., 2017; Saha et al., 2008) when changing the hip angle. For example, when approaching a drop in ground level during walking, an upward rotation of the trunk during the step-down would increase the distance between CoM and foot and thus limit changes in CoM height which in turn would likely lead to reduced changes in kinetic behaviour. Humans might exploit this mechanism that in some way resembles the ability of small birds to adjust their zig-zag-like configured legs when coping with ground level perturbations (Birn-Jeffery and Daley, 2012; Birn-Jeffery et al., 2014; Müller et al., 2016). In this sense, we expect that the upper body might be transformed into an active component of the human locomotor system in trunk-flexed walking.

Studies of perturbed human locomotion often focus on gait dynamics when either posture, surface or movement is individually perturbed. A study by Saha et al. (2008) revealed that dynamic balance during walking with 25° and 50° sagittal trunk flexion in able-bodied participants is achieved by adjusting lower limb kinematics to more crouched configurations. They reported a higher GRF and loading rate during weight acceptance phase and a lower GRF during pre-swing phase. In a recent study, Aminiaghdam, et al. (2017) found that proceeding to a horizontal trunk configuration in humans caused similar dynamic intra-limb asymmetries in leg function as compared with birds. Such asymmetries, found to be necessary for maintaining dynamic balance in pronograde gait (Andrada, et al., 2014), were characterised by a reduction of the effective leg (connecting hip to centre of pressure) length and the GRF in the pre-swing phase as compared to the weight acceptance phase (Andrada et al., 2014; Aminiaghdam et al., 2017). Comparing human and avian running on uneven ground, Müller et al. (2016) reported that despite striking morphological disparities these species share some common kinematic behaviour (i.e. leg angle and leg length) while negotiating changes in ground level. For walking on uneven ground, when human walkers encounter a drop, they modulate their GRF kinetics proportional with the drop height not only in the perturbation step, but also in the approach step to the perturbation (Müller, et al., 2014). However, the quality and quantity of the kinetic and kinematic adaptations or reactions to external perturbations are context-specific (Müller, et al., 2014; van der Linden, et al. 2009, 2007). While these studies have analysed human walking with various trunk configurations or adaptive and reactive kinetic mechanisms in pre-perturbation and perturbation contacts and made comparisons with avian locomotor behaviour, to our knowledge, kinetic and kinematic adaptations when stepping down (perturbation) and in pre- and post-perturbation steps with different bent postures have not been investigated yet.

In this study, we investigate kinetic characteristics of the GRF during the stance phase across three steps in uneven ground, i.e. in the perturbation and pre- and post-perturbation steps, as a function of trunk orientation compared with unperturbed step in level ground. Trunk-flexed gaits and accommodation of changes in ground levels are expected to lead to posture- and step-specific main effects on GRF characteristics as compared to the upright walking and level walking, respectively. We hypothesise a systematic change in patterns of GRF as a function of walking posture within each step, however walking with bent postures would demonstrate reduced kinetic adaptations across steps in uneven ground relative to the unperturbed level ground step as altering the trunk angle might facilitate kinematic adaptations to changes in ground level. For example, we expect that the aligned effects of trunk flexed gait and step down on the first GRF peak in the perturbation step and on the second GRF peak in the pre-perturbation step do not simply add up to avoid excessive loads and falling down, respectively.

## RESULTS

The data analyzed comprises 768 trials with a total of 2304 step cycles. All healthy young participants on every trial were successful in maintaining their stability (no falls) while traversing the travel path with and without drop. Table 1 summarises posture×step interactions and the main effects of posture and step.

**Table 1.**
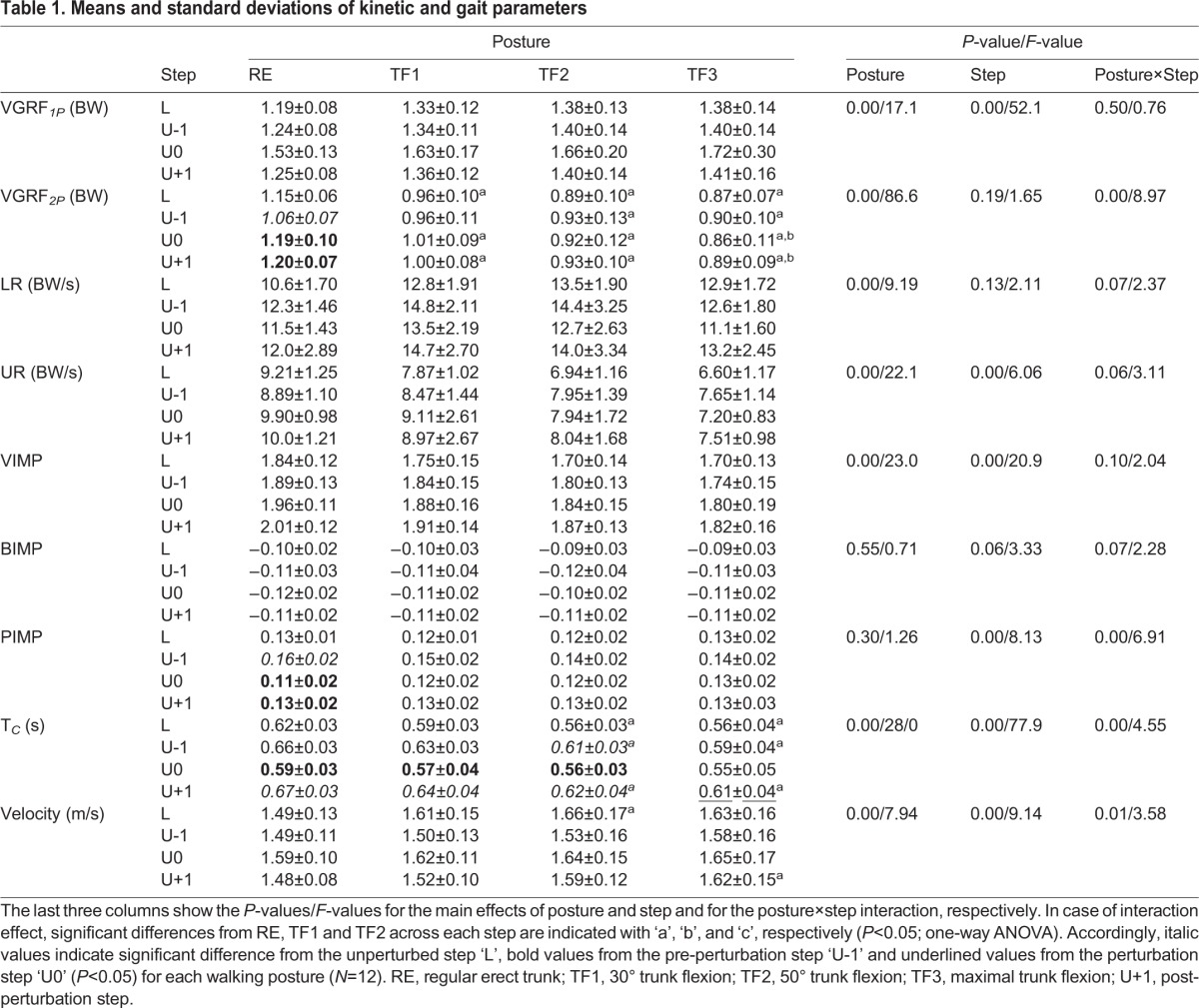
**Means and standard deviations of kinetic and gait parameters**

### Main effects of posture

**Fig. 1.**
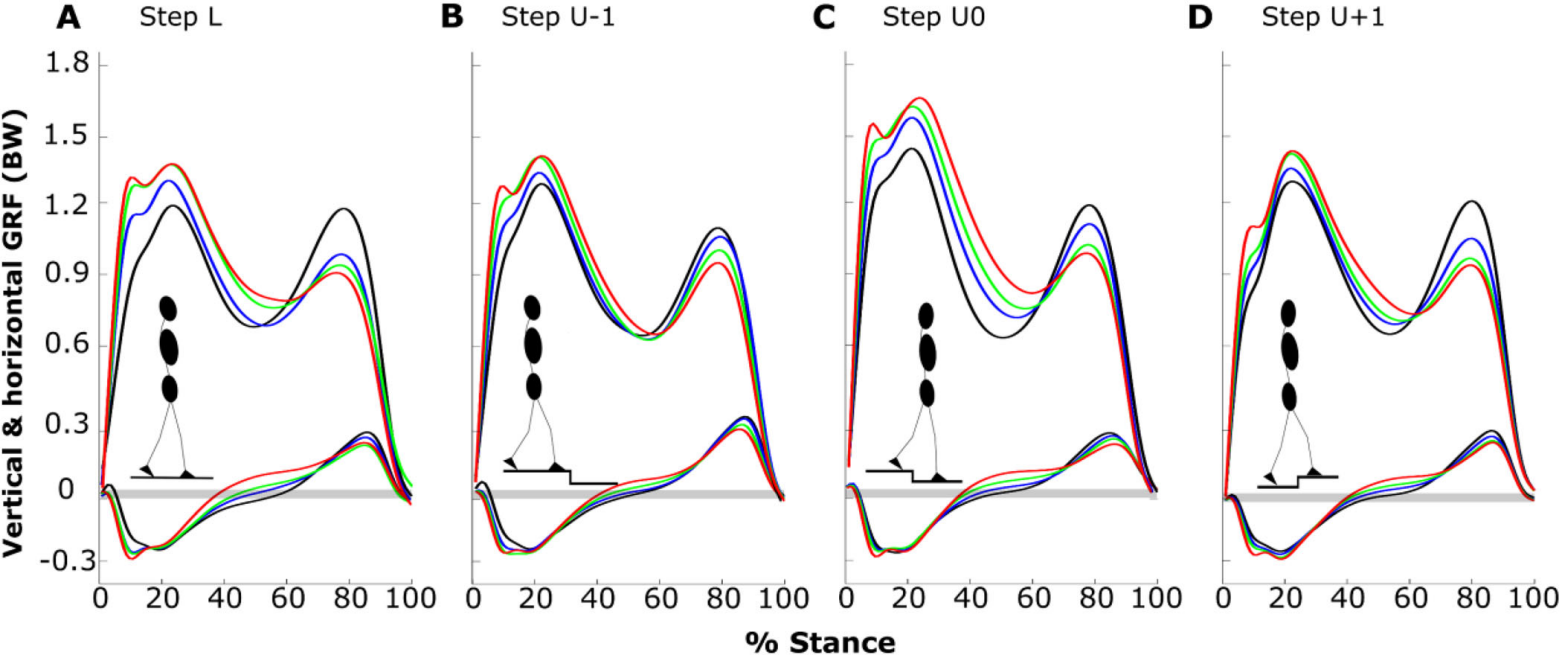
**Ground reaction forces (GRF) for different walking conditions.** Shown are ensemble-averaged horizontal and vertical GRFs [normalized to participant body weight (BW)] during unperturbed level step (L, A), pre-perturbation step (U-1, B), perturbation step (U0, C) and post-perturbation step (U+1, D) for RE (black), TF1 (blue), TF2 (green) and TF3 (red) gaits during the stance phase (*N*=12). The contact time is normalized to 100%.

**Fig. 2.**
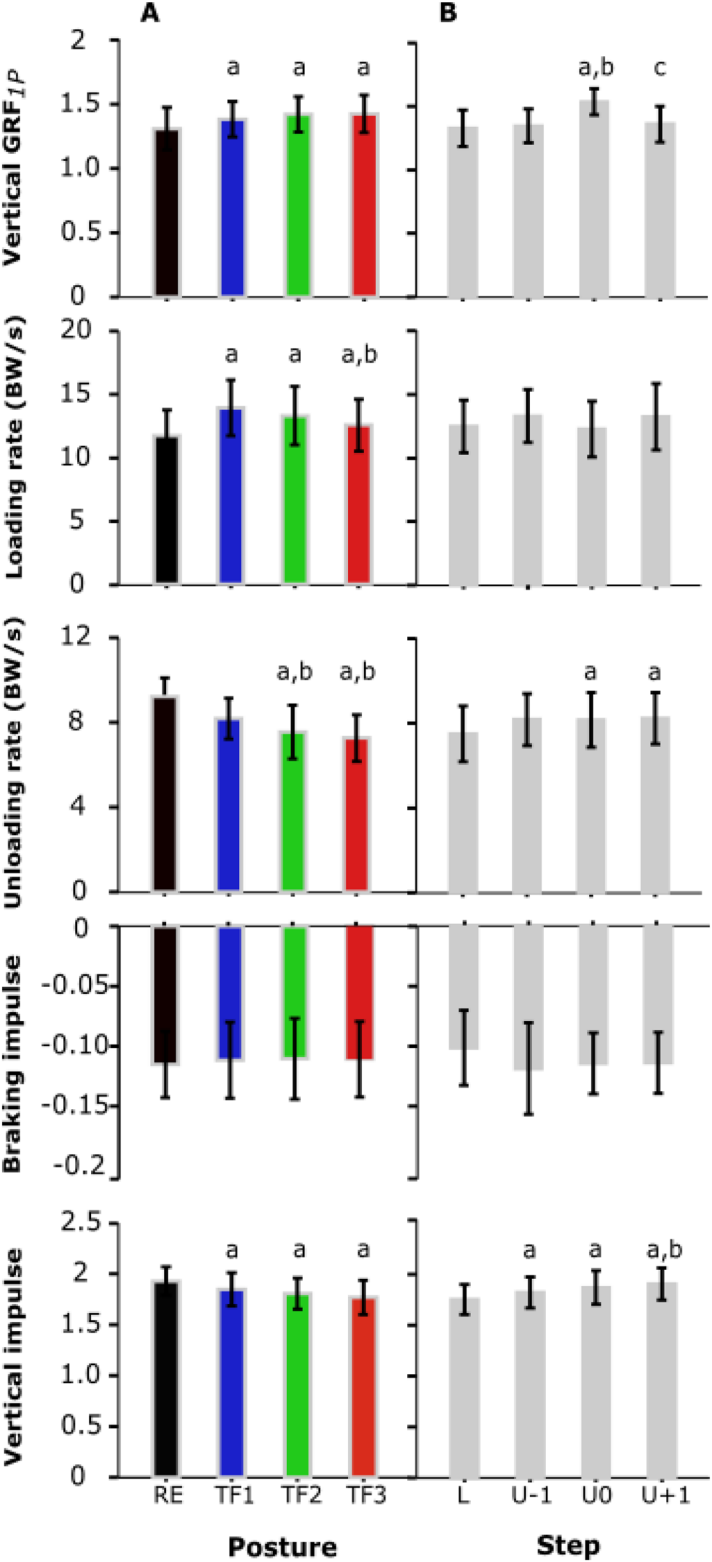
**Main effects of posture and step.** Shown are the mean and standard deviations (error bars) for the main effects of posture (A) and step type (B) on the first peak of the vertical GRF, loading rate, unloading rate, braking impulse and vertical impulse (*N*=12). Significant differences from RE, TF1 and TF2 as well as from ‘L’, ‘U-1’, and ‘U0’ are indicated with ‘a’, ‘b’, and ‘c’, respectively (*P*<0.05; one-way ANOVA). RE (black), regular erect trunk; TF1 (blue), 30° trunk flexion; TF2 (green), 50° trunk flexion; TF3 (red), maximal trunk flexion; L, unperturbed level step; U-1, pre-perturbation step; U0, perturbation step; U+1, post-perturbation step.

With more sagittal trunk flexion (averaging over the steps), the unloading rate (UR) decreased and, less clearly, the first peak in the GRF (VGRF_*1P*_) increased, while the vertical impulse (VIMP) decreased (Fig. 1 and Fig. 2A, Table 1). More specifically, comparing TF3 gait with regular erect (RE) gait, UR decreased by 21% [to 9.19±0.88 (mean±standard deviation)], VGRF_*1P*_ increased by 14% (to 1.48±0.18), and VIMP decreased by 8% (to 1.77±0.16) (Fig. 1 and Fig. 2A). For trunk-flexed gaits the loading rate (LR) was generally higher than in RE gait, and the highest LR was observed during walking with 30° sagittal trunk flexion (TF1) gait (13.8±2.17) with an increase of ∼19% relative to RE gait (Fig. 2A). By contrast, increased sagittal trunk flexion did not lead to a change in the braking impulse across gaits (Fig. 2A).

### Main effects of step

Only VGRF_*1P*_, UR and VIMP showed main effects (Table 1) when averaging over the postures, and most effects occurred in the perturbation step (Fig. 2B). Relative to the level step ‘L’, VIMP increased by 4% (to 1.82±0.15), 7% (to 1.87±0.16) and 9% (to 1.90±0.15) for pre-perturbation step ‘U-1’, perturbation step ‘U0’, and post-perturbation step ‘U+1’, respectively, VGRF_*1P*_ increased by 23% (to 1.63±0.10) for ‘U0’, and UR increased by 9% (to 8.13±1.29) and 10% (to 8.21±1.20) for ‘U0’ and ‘U+1’, respectively (Fig. 2B).

### Interaction effects posture by step

**Fig. 3.**
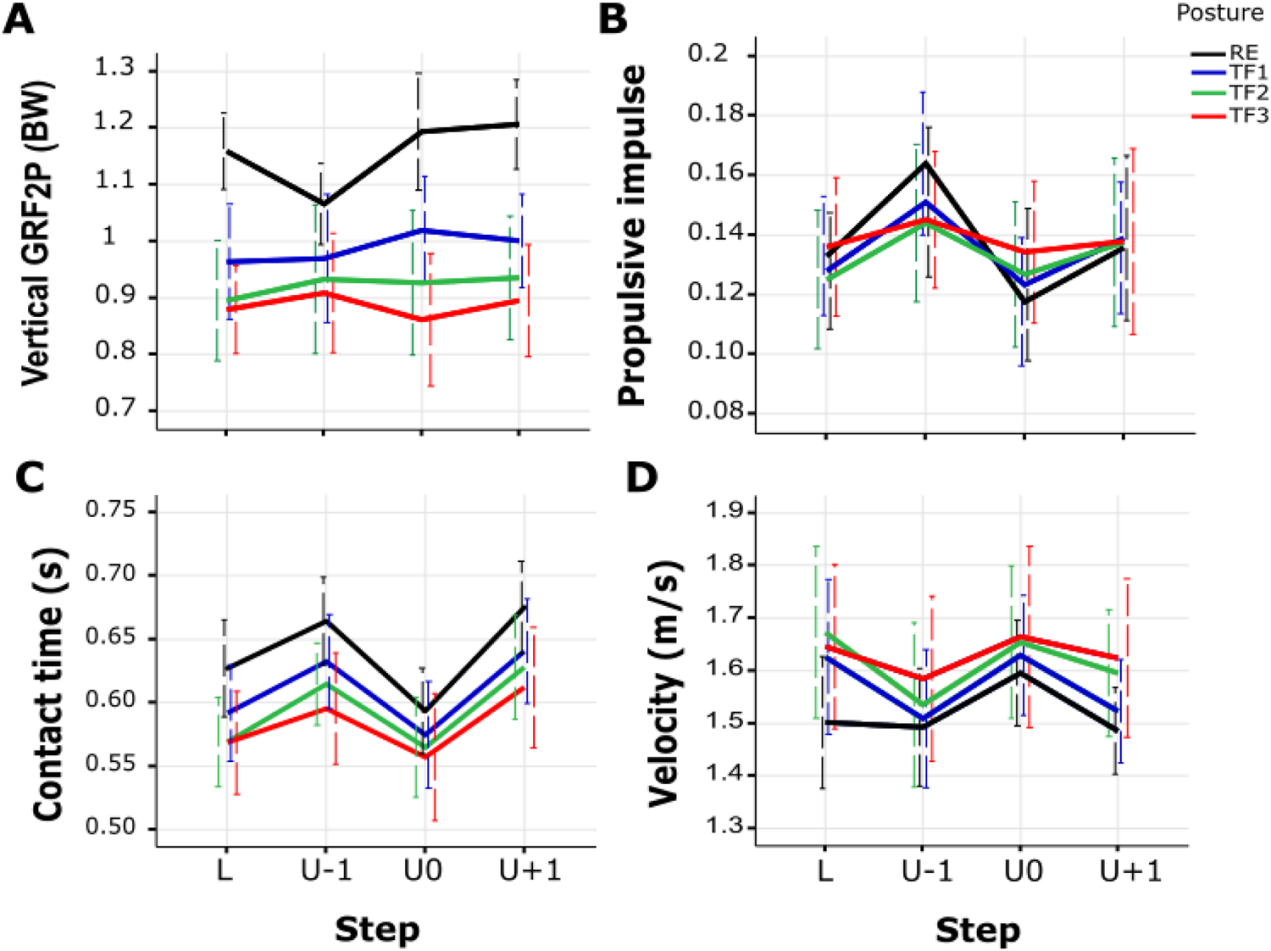
**Posture×step interaction.** Shown are posture×step interactions on the second peak of vertical GRF (A), propulsive impulse (B), contact time (C) and velocity (D) (*N*=12). Error bars indicate ±standard deviation. RE, regular erect trunk; TF1, 30° trunk flexion; TF2, 50° trunk flexion; TF3, maximal trunk flexion; L, unperturbed level step; U-1, pre-perturbation step; U0, perturbation step; U+1, post-perturbation step.

Step-dependent effects of posture were detected for the second peak of the vertical GRF (VGRF_*2P*_), propulsive impulse (PIMP), contact time (T_C_) and velocity (Table 1). While in RE gait, VGRF_*2P*_ first decreased in ‘U-1’ and then increased in ‘U0’, this pattern gradually reversed with increasing trunk flexion (Fig. 3A). Moreover, the pronounced differences in propulsive impulse between steps for RE gait diminished with increasing trunk flexion (Fig. 3B), and differences in contact time decreased in ‘U0’ (Fig. 3C). While velocity remained constant in steps ‘L’ and ‘U-1’ in RE gait, it decreased in trunk-flexed gaits (Fig. 3D).

RE gait showed step-dependent effects for all variables exhibiting interaction except for velocity (Table 1). In contrast, trunk-flexed gaits demonstrated step-dependent effects only for T_*C*_ (Table 1). No posture-dependent effects were observed for PIMP and only two for velocity (Table 1). Trunk-flexed gaits consistently showed posture-dependent effects compared with RE gait for VGRF_*2P*_ (decrease) and less consistently for T_*C*_ (decrease, no effect for TF1) (Fig. 3A,C, Table 1). Notably, except for two posture-dependent effects on VGRF_*2P*_ during steps ‘U0’ and ‘U+1’ in TF3 gait, no effects were found within trunk-flexed gaits (Table 1). T_*C*_ and velocity did not show posture-dependent effects in the perturbation step ‘U0’ (Fig. 3C,D, Table 1).

## DISCUSSION

In this study, the adaptive kinetic behaviour of able-bodied walking while negotiating uneven ground with altered trunk orientations was investigated. A systematic change of the patterns of GRF as a function of walking posture and step type was observed (Fig. 1). We found step-dependent effects of posture for the second peak of the vertical GRF, propulsive impulse, contact time and velocity (Fig. 3, Table 1). For these variables, simple main effect analysis showed that walking with trunk-flexed gait was associated with reduced changes across steps in uneven ground (perturbation, pre- and post-perturbation steps) compared with upright walking (Table 1). Main effects of posture and step categories on able-bodied walking were observed in the majority of cases, indicating posture- and step-specific GRF characteristics (Fig. 2). In the following paragraphs, the individual main effects of posture and step as well as their interaction effects on the gait kinetics will be discussed in detail.

### Posture-dependent kinetic behaviour

Studies on level walking with a trunk-flexed gait have shown that the alteration of trunk kinematics in sagittal plane leads to compensatory kinematic adjustments in lower limbs, which in turn causes changes in the gait kinetics (Saha et al., 2008; Aminiaghdam et al., 2017). Accordingly, our results highlight that the GRF profile varies with an increase of sagittal trunk flexion, regardless of ground condition (Fig. 1). The vertical GRF profile tended to be more asymmetric, i.e. greater forces during weight acceptance and attenuated forces during push-off as the trunk leans far forward (Fig. 1). Such right-skewed profiles of vertical GRF exhibited higher weight acceptance loads associated with higher loading rates, a lower push-off associated with lower unloading rates and lower vertical impulses (Figs 1 and 3, Table 1). Such behaviour is consistent with a simple effective leg model of spring-and-damper-in-series (Aminiaghdam et al., 2017). In that study, we have shown that the damper right-skews the GRF by increasing forces after touchdown and decreasing the forces at toe-off leading to an earlier lift-off. Surprisingly, despite remarkable disparities in the morphology of segmented legs between human and bird, experimentally induced pronograde locomotion in human yields kinematic and kinetic effective leg behaviour comparable to those found in birds (Aminiaghdam et al., 2017).

Increased loading rates and lower unloading rates have been found in dysfunctional gait in many studies, for example in patients with Down syndrome (Wu and Ajisafe, 2014), with knee osteoarthritis (Farrokhi et al., 2015; Silva Dde et al., 2015), in elderly female individuals during stair ascent (Hamel et al., 2005) or obese individuals (Pamukoff et al., 2016), and in loaded gait while carrying a back pack (Park et al., 2016). Trunk orientation causes similar effects (Fig. 2A). These changes reflect adaptations of the gait pattern. For example, in both animals and humans, a swift transition from stance to swing is actuated by unloading at higher rates during pre-swing phase (Grillner, 1985; Pearson et al., 1992; Pang and Yang, 2000). Furthermore, the active ankle push-off is responsible for initiating the leg swing in humans (Lipfert et al., 2014). In trunk-flexed walking, this push-off is impaired as judged from the lower VGRF_*2P*_, and the unloading rate is lower (Figs 1 and 3A) than in RE gait. Trunk kinematics therefore may be considered as a significant criterion for clinicians not only in the assessment of dysfunctional gait, but also in the design, development and monitoring of the progression of rehabilitation regimes.

Owing to a shorter contact time, the vertical impulse is diminished in the trunk-flexed gaits compared with RE gait (Fig. 2A). This requires a faster swing phase and a higher cadence to support body weight. Such a decrease in vertical impulse has also been observed during level walking while adopting the same bent postures (Aminiaghdam et al., 2017). Moreover, in accordance with our previous study on trunk-flexed level walking, altered trunk kinematics yielded no change in braking impulse (Fig. 2A, Table 1). There, we demonstrated that an increased sagittal trunk flexion leads to a shorter braking phase relative to the propulsive phase and a greater braking peak force (Aminiaghdam et al., 2017). Hence, the unchanged braking impulse in uneven walking might be the consequence of a combination of a rapid deceleration of the body mass and a greater braking force.

### Step-dependent kinetic behaviour

When human walkers become aware of changes in the ground level, e.g. a drop, they adjust their locomotor strategies in the step before the perturbation (Müller and Blickhan, 2010; Müller et al., 2012, 2014, 2016). For the main effect of the step type, our results revealed a significant effect in the pre-perturbation step only in case of the vertical impulse (4% increase relative to level step, Fig. 2B).

The longer flight time associated with the step down led to a greater VGRF_*1P*_ (16% increase relative to level step) in the perturbation step ‘U0’. The greater vertical impulse (9% increase relative to level step) in this step is largely due to a greater vertical GRF as contact time did not significantly extend relative to the level step (Fig. 2B, Table 1). Human walkers with regular upright posture negotiate visible and camouflaged drops in ground using the same strategy, i.e. a shorter contact time and a longer double support (Müller et al., 2014). The observed higher unloading rate in ‘U0’ (7% increase relative to level step, Fig. 2B) may be due to an earlier landing after a shorter swing phase of the contralateral limb on an elevated surface in the subsequent step along with a slight increase of the vertical GRF at the end of the stance phase (Table 1).

A greater vertical impulse (9% increase relative to level step, Fig. 2B) in post-perturbation step ‘U+1’ is the result of a significantly longer contact time which is required for the elevation and propulsion of the CoM after the drop (Fig. 2B, Table 1). Moreover, participants were able to produce a greater push-off at the end of the stance phase reflected in increased second peak of the vertical GRF, which led to higher unloading rates (10% increase relative to level step, Fig. 2B).

### Interaction of posture and step

**Fig. 4.**
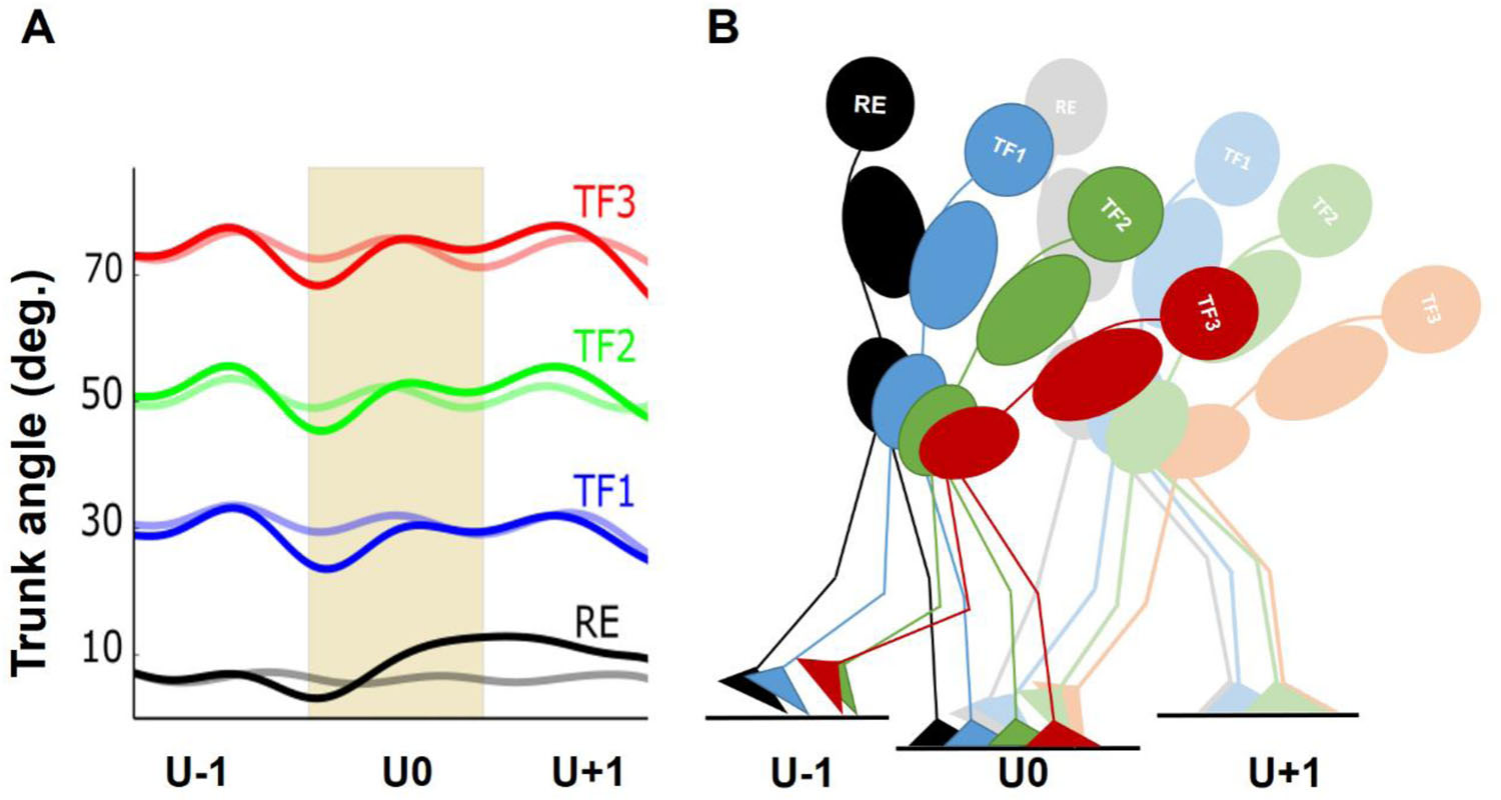
**Trunk kinematics and human locomotion diagram.** (A) The trunk kinematics in the sagittal plane across three level (pale lines) and three uneven steps (solid lines) with regular erect (RE, black), 30° trunk flexion (TF1, blue), 50° trunk flexion (TF2, green), maximal trunk flexion (TF3, red) postures. The shaded area, the second step across two setups, separates pre- and post-perturbation steps. (B) Side view of the instrumented walkway with three consecutive force plates denoted by U-1 (pre-perturbation step), U0 (perturbation step) and U+1 (post-perturbation step). The second force plate (drop) was lower by 10 cm in walking on uneven ground.

Step-specific effects of gaits with different trunk orientations were observed for VGRF_*2P*_, propulsive impulse, contact time and velocity (Table 1). As hypothesised, among these variables we found reduced kinetic adaptations in trunk-flexed gaits across steps in uneven ground when compared with RE gait (Table 1). This was in agreement with our hypothesis that, in trunk-flexed gaits, the trunk could be utilised to negotiate changes in ground level by straightening during step down. In fact, such straightening is evident in Fig. 4A. In contrast with one of our hypotheses that aligned effects of trunk-flexed gait and step-down on the first GRF peak in the perturbation step do not simply add up to avoid excessive loads, interaction was not strong enough to yield a significant effect across all steps.

As for the two kinetic parameters exhibiting interaction, an increase of trunk flexion led to a decrease in the VGRF_*2P*_ but no changes in propulsive impulse across gait postures. In comparison to RE gait in the step ‘U0’, for example, TF3 gait exhibited 28% decrease in the VGRF_*2P*_ (Figs 1 and 3A, Table 1). Owing to an earlier toe-off at a steeper effective leg angle, the trunk-flexed gait in human and birds is associated with more flexed leg joints and decreased effective leg length at toe-off compared with touchdown (Grillner, 1985; Pearson et al., 1992; Pang and Yang, 2000; Andrada et al., 2014; Aminiaghdam et al., 2017). In fact, such kinematic behaviour yields an inefficient push-off reflected in low VGRF_*2P*_. Furthermore, a combination of a longer propulsive phase and a lower magnitude of the propulsive force in trunk-flexed gaits resulted in no significant difference in propulsive impulse from normal walking (Fig. 3B, Table 1). In contrast with RE gait, step-dependent effects of posture in trunk-flexed gaits on VGRF_*2P*_ and propulsive impulse were not observed (Table 1).

For the gait parameters, i.e. contact time and velocity, simple main effects showed that with increasing deviation of the trunk from upright, they become shorter and faster, respectively. Surprisingly, adaptations in the pre-perturbation step led to approximately the same contact time and speed regardless of trunk orientation in the perturbation step (Fig. 3C,D, Table 1). Moreover, walking with different trunk orientations yielded no significant change in velocity across steps. This was reflected in braking and propulsive impulses where also no changes were observed during various gait conditions across steps, except in the approach step to the drop where propulsive impulse increased in RE gait (Fig. 3B, Table 1). As a result, individuals performed steady state gaits at each trunk posture. However, except for TF3, in other gaits human walkers performed the post-perturbation step with a longer contact time.

### Conclusion

Expanded analysis of walking across uneven ground revealed that GRF parameters were more consistent for trunk-flexed gaits. Pre-adaptations were more pronounced in the approach step to the drop in regular erect gait. This observation is tentatively explained with the role of the trunk. In contrast with walking with upright trunk, in trunk-flexed gaits the trunk may be used in a compensatory way during the step-down to accommodate changes in ground level by adjusting its angle leading to reduced variations in CoM height during traversing uneven ground. Exploitation of this mechanism would resemble the ability of small birds in adjusting their zig-zag-like configured legs to cope with large ground level perturbations.

## MATERIALS AND METHODS

### Participants

Six males and six females (mean±s.d.; age 26±3.35 years, height 169.75±7.41 cm, mass 65.08±8.07 kg), free from health problems that could affect their walking pattern and trunk motion, were recruited for this study. A consent form was signed by each participant before participation. The experimental protocol was approved by the local Ethics Committee of the Friedrich Schiller University Jena (3532-08/12) and carried out in accordance with the Declaration of Helsinki.

### Experimental design and measurements

Data collection was conducted at the Biomechanical Laboratory of the Sports Institute within Friedrich Schiller University Jena. All trials were recorded with eight cameras (240 Hz) by a 3D infrared system (MCU1000, Qualisys, Gothenburg, Sweden) and synchronised by using the trigger of Kistler soft- and hardware. Three consecutive force platforms (9285BA, 9281B, 9287BA, Kistler, Winterthur, Switzerland) embedded in the middle portion of a 12 m-long walkway and sampled at 1000 Hz. 21 markers (spherical retro-reflective surface, 14 mm) defined a 13-body segment model. The markers were placed on the following bony landmarks: fifth metatarsal heads, lateral malleoli, lateral epicondyles of femurs, greater trochanters, anterior superior iliac spines, posterior superior iliac spines, L5-S1 junction, lateral humeral epicondyles, wrists, acromioclavicular joints, seventh cervical spinous process and middle of the forehead (Aminiaghdam et al., 2017).

Participants were asked to walk at their self-selected normal walking speed under four trunk flexion conditions (with no restriction on the arm movements) across two experimental ground conditions involving a level walkway and a walkway with a 10-cm drop: self-selected regular erect trunk alignment (RE), 30° (TF1), 50° (TF2), and maximal trunk flexion (TF3) (Fig. 4A). One height-variable force plate at the site of the second step and two ground-level force plates at the site of the first and third steps were set (Fig. 4B). After walking on the unperturbed uniform track, the variable-height force plate was lowered by 10 cm and participants walked along the uneven walkway. Trunk flexion was achieved by bending from the hips, which allows the most consistent trunk posture among participants (Saha et al., 2008; Aminiaghdam et al., 2017). Under such definition, the TF3 constituted the maximum amount of trunk flexion that the participants could adopt while walking (Fig. 4). Trunk angle was defined by the angle sustained by the line connecting the midpoint between the L5–S1 junction (L5) and the seventh cervical spinous process (C7) with respect to the vertical axis of the lab coordinate system (Müller et al., 2014; Aminiaghdam et al., 2017). Trunk angles were compared visually with adjustable-height cardboard templates by a second examiner prior to performing of each trial and during gait along the walkway for TF1 and TF2. For TF3, there was no comparison. The templates, drawn with angles displaying target trunk flexion angles TF1 and TF2, were hung on a wall parallel to the walkway: one at the beginning and the other one in the middle of the walkway (Saha et al., 2008; Aminiaghdam et al., 2017). Practice trials were permitted to allow participants to accommodate to the locomotion conditions and to secure step onto the force plates. Five out of twelve participants were identified to have a dominant left leg. To eliminate the influence of the dominant leg (Sadeghi et al., 2000), we instructed all participants to hit force plates in left-right-left sequence (Müller et al., 2014). Due to organisational reasons, level and uneven setups as well as repetitions of trunk orientations were not randomised, but the sequence of flexed trunk orientations were randomised per participant. The participants accomplished eight trials per condition in which each foot stepped on a single force plate.

The following parameters of interest were determined across each step: the first peak of the vertical GRF (VGRF*_1P_*) and the second peak of the vertical GRF (VGRF*_2P_*); loading rate (LR) and unloading rate (UR) as the slope of vertical GRF between initial heel strike and the VGRF*_1P_* and between the VGRF*_2P_* and toe-off, respectively; vertical impulse (VIMP) by integrating the vertical GRF, braking impulse (BIMP) and propulsive impulse (PIMP) by integrating the anterior–posterior GRF over the time that the force was oriented in the posterior and anterior directions, respectively, and normalised to the product of body weight and the square root of the quotient of leg length and gravity (Hof, 1996); contact time (T*_C_*) as the time duration between the initial heel strike and toe-off; gait velocity as mean of horizontal velocity of the L5 marker between the initial heel strike and toe-off. For kinetic analysis, GRF was normalised to participant body weight (BW). A vertical GRF threshold of 0.03 BW was used to determine the instants of the initial heel strike and the toe-off at each step.

### Data processing and statistics

Kinetic and kinematic data of all successful trials were analysed using custom written Matlab (Mathworks Inc., MA, USA) code. The raw coordinate data were filtered using a fourth-order low-pass, zero-lag Butterworth filter with 12 Hz cut-off frequency (Aminiaghdam et al., 2017). For our normally distributed data, two-way repeated-measures ANOVAs were implemented with SPSS (IBM SPSS Statistics 22, Armonk, NY, USA) using two within-participants factors: (1) step category (unperturbed step ‘L’ during level walking; pre-perturbed ‘U-1’, perturbed ‘U0’ and post-perturbation ‘U+1’ steps during uneven walking), and (2) postures (RE, TF1, TF2 and TF3). The posture×step interaction was evaluated for each dependent variable of interest. Post hoc comparisons were performed using Bonferroni. A *P*-value of *P*<0.05 was considered as statistically significant in all cases. In case of a significant interaction, simple main effects were used to compare walking postures across each step and steps while walking with each posture. In case of a non-significant interaction, the main effects of the posture (averaging across the steps) and the step (averaging across the postures) were evaluated for each variable of interest using one-way ANOVA and post hoc comparisons.
